# Editorial: Metal/Covalent Organic Frameworks and Their Derivatives for Electrochemical Energy Applications

**DOI:** 10.3389/fchem.2020.617614

**Published:** 2020-12-11

**Authors:** Jian-Gan Wang, Fei Xu, Yu Zhang

**Affiliations:** ^1^State Key Laboratory of Solidification Processing, Center for Nano Energy Materials, School of Materials Science and Engineering, Northwestern Polytechnical University and Shaanxi Joint Lab of Graphene (NPU), Xi'an, China; ^2^Department of Inorganic Chemistry, Technische Universität Dresden, Dresden, Germany; ^3^School of Mechanical and Power Engineering, East China University of Science and Technology, Shanghai, China; ^4^Department of Chemical and Biological Engineering, Hong Kong University of Science and Technology, Kowloon, Hong Kong

**Keywords:** metal-organic frameworks, covalent organic frameworks, supercapacitors, Li-Sulfur batteries, water splitting, fuel cells

Metal-organic frameworks (MOFs) and covalent organic frameworks (COFs) have gained extensive research interest in a myriad of electrochemical energy fields. In this Research Topic, we have collected some original research articles and a comprehensive review based on the MOFs/COFs and/or their derivatives for the use in Li-sulfur batteries, supercapacitors, water splitting, as well as fuel cells. These results demonstrate the promising applications of MOFs/COFs and/or their derivatives toward electrochemical energy communities. Specifically, Hua et al. reported a freestanding V-doped CoP nanosheet array supported on the carbon cloth by a facile cation exchange/phosphorization treatment on a Co-MOF precursor. The as-prepared V-doped CoP could serve as a highly efficient hydrogen evolution reaction (HER) catalyst in both acidic and basic media. It was revealed that the three-dimensional nanoarray architecture enabled a large electrochemical active surface area to expose more active sites for boosting the HER kinetics. The study also demonstrated the great effectiveness of hetero-atoms doping to enhance the intrinsic catalytic activity of CoP by tuning the electronic properties. Impressively, small overpotentials of 88 and 98 mV were required to afford a current density of 10 mA cm^−2^ in 0.5 M H_2_SO_4_ and 1 M KOH electrolytes, respectively. He et al. employed an interesting “One-for-All” design strategy to purposely prepare hierarchically nanoporous carbon and ZnCo_2_O_4_ particles from a single bimetallic Zn/Co-MOF precursor. The as-synthesized carbon and metal oxide samples were utilized as the negative and positive electrodes to construct asymmetric supercapacitors. Notably, the unique nanoporous structure could provide sufficient active interfaces for delivering high specific capacity. The asymmetric configuration further extends the working voltage window to as high as 1.45 V, thus achieving a high energy density of 28.6 Wh/kg at a power density of 100 W/kg and good cycling stability of 87.2% after 5,000 charge-discharge cycles. Xu et al. reported the design of amide functionalized covalent triazine frameworks (CTFs) as host materials in lithium-sulfur batteries and for liquid-phase dye adsorption. Amide functionalized CTFs (CTF-PO71) were prepared with pigment PO71 bearing amide group as monomer under ionothermal condition with ZnCl_2_ as solvent and catalyst. The pore structure and functional groups can be controlled by the amount of ZnCl_2_ to monomer ratio. Afterwards, the effect of pore structure and functional groups on the adsorption-based performances were investigated in detail. The results showed that significant improvement of lithium-sulfur battery performance was achieved compared to non-functionalized CTF, benefitting from the promoted entrapment of polysulfide results from cooperative effect of physical confinement by high porosity and the chemisorption resulting from functional groups in CTF-PO71 networks. Meanwhile, dye adsorption performance revealed the crucial role of amide functional groups for chemical interaction and the pore structure for facile access and selective adsorption toward specific dye molecules. As a core component of proton exchange membrane fuel cells (PEMFCs), proton exchange membrane suffers from significant performance challenges prior to the practical implementation. MOFs can accommodate a large number of water molecules and acidic groups due to their high specific surface area; thus providing sufficient receptors for proton transfer. Liu et al. provided an urgent overview of the recent progress of MOF modified PEM for PEMFCs. In particular, this review presents a detailed discussion on the effect of different types of MOFs, including UiO-series, MIL-series, ZIF-series, and others, on the performance of PEMFCs. Finally, the challenges and perspectives in this field were provided.

## Author Contributions

J-GW: writing—review and editing. FX: writing—editing. YZ: writing. All authors contributed to the article and approved the submitted version.

## Conflict of Interest

The authors declare that the research was conducted in the absence of any commercial or financial relationships that could be construed as a potential conflict of interest.

